# Time mental accounting and novice employees’ intertemporal choices: mediating effects of time management disposition and future self-continuity

**DOI:** 10.3389/fpsyg.2025.1743618

**Published:** 2026-01-27

**Authors:** Jie Liu, Mingwei Zhang, Tao Yu, Lidong He, Yuzhen Wu, Xiaofu Pan

**Affiliations:** 1 College of State Governance, Southwest University, Chongqing, China; 2 Faculty of Psychology and Educational Sciences, Ghent University, Ghent, Belgium; 3 College of Life Sciences, Southwest University, Chongqing, China; 4 School of Marxism, Chongqing University of Chinese Medicine, Chongqing, China; 5 State Key Laboratory of Ultrasound in Medicine and Engineering, Chongqing Medical University, Chongqing, China

**Keywords:** future self-continuity, intertemporal choice, novice employees, time mental accounting, time management disposition

## Abstract

**Introduction:**

This study examined how time mental accounting relates to intertemporal decision-making among novice employees and whether this relationship operates through time management disposition (TMD) and future self-continuity (FSC), drawing on Conservation of Resources theory.

**Methods:**

A nationwide sample of 597 early-career employees in China completed validated measures of time mental accounting (loss aversion, mental budgeting, and flexibility), TMD, and FSC, as well as eight binary intertemporal choice tasks contrasting smaller-sooner versus larger-later rewards. Correlations and structural equation modeling with bias-corrected bootstrapping were conducted, controlling for age, gender, and education.

**Results:**

Loss aversion and mental budgeting were positively associated with TMD and FSC and negatively associated with smaller-sooner choices, whereas flexibility showed no significant associations. Mediation analyses indicated that the effects of time mental accounting facets on intertemporal choice were transmitted indirectly via TMD and FSC, with non-significant direct effects when mediators were included.

**Discussion:**

The findings support a dual-path mechanism whereby structured time allocation and stronger future self-identity function as complementary resources that reduce short-term bias in novice employees’ intertemporal decisions.

## 1 Introduction

Intertemporal choice—the decision between smaller-sooner rewards and larger-later rewards—constitutes a fundamental topic in behavioral economics and psychology (Frederick et al., 2002). Extensive evidence shows that people systematically discount the future, preferring immediate gratification even when delayed rewards yield greater utility (Peters and Büchel, 2011; Burghoorn et al., 2025). This present-biased tendency influences financial, health, and consumer decisions alike (Tuyizere et al., 2024). Within organizations, employees face comparable trade-offs—whether to pursue short-term performance goals or invest in long-term growth. Yet most research remains confined to laboratory or consumer contexts, overlooking how such temporal dilemmas unfold in workplaces where time is not merely chronological but socially and psychologically embedded. Consequently, little is known about how individuals allocate and value their limited temporal resources when balancing competing short- and long-term objectives.

This gap is particularly pronounced among novice employees—those entering the workforce for the first time—who often struggle to reconcile daily job demands with career development. Prior research indicates that younger or less experienced individuals exhibit weaker self-regulation and future orientation during major life transitions (De Ridder et al., 2012). Their limited experience, cognitive capacity, and social capital heighten vulnerability to present bias and reactive decision-making (De Vos et al., 2020). Although studies on time management and goal-setting have addressed behavioral regulation, they rarely intersect with intertemporal choice frameworks (Aeon and Aguinis, 2017; Claessens et al., 2007). Moreover, temporal preferences are often treated as stable traits, disregarding how cognition and context dynamically shape time-related decisions (Soman, 2001; Zauberman and Lynch, 2005). This theoretical fragmentation obscures how cognitive framing, behavioral regulation, and motivational identity jointly shape workplace decision-making—especially how individuals mentally categorize, value, and trade off their temporal resources.

Addressing this gap is both theoretically and practically essential. Early intertemporal decisions set cumulative trajectories influencing career sustainability and wellbeing. Inability to balance short- and long-term demands fosters short-termism that undermines personal and organizational development. The Conservation of Resources (COR) theory offers an integrative lens for these dynamics. COR posits that individuals seek to acquire, protect, and invest valued resources, with loss or depletion triggering stress and performance decline (Hobfoll, 1989). Within this framework, time represents a finite and nonrenewable resource, while self-regulatory capacity functions as a psychological asset that can be strengthened through deliberate management (Halbesleben et al., 2014). Conceptualizing time as a cognitive resource clarifies why early-career employees—whose reserves are limited—must strategically invest temporal and psychological assets to prevent “loss spirals” and sustain adaptive functioning.

Drawing on COR theory, this study introduces Time Mental Accounting (TMA) as a cognitive mechanism by which individuals allocate, budget, and safeguard temporal resources. Originally proposed to explain how people mentally organize financial outcomes (Thaler, 1980, 1999), mental accounting theory has since been extended to time. TMA refers to the cognitive process of structuring, valuing, and evaluating trade-offs among temporal segments (Soman, 2001; Jachimowicz et al., 2018). We further propose a dual-path model in which TMA influences intertemporal choice indirectly through Time Management Disposition (TMD)—the behavioral enactment of structured time use—and Future Self-Continuity (FSC)—the motivational linkage between present and future selves. Integrating these constructs connects cognitive framing, behavioral regulation, and motivational identity into a unified framework of intertemporal decision-making in early-career contexts.

To ground this framework, we integrate insights from behavioral economics, temporal psychology, and self-regulation theory. Behavioral economics conceptualizes intertemporal choice as a trade-off between immediate and delayed rewards; temporal psychology explains how people mentally represent and allocate time; and self-regulation theory highlights the alignment of cognitive control and sustained motivation in goal pursuit (Aeon and Aguinis, 2017). Together, these perspectives yield a comprehensive account of how individuals allocate, conserve, and invest temporal resources under competing demands. Within this integrative model, TMA, TMD, and FSC jointly explain sustainable career development and employability (De Vos et al., 2020; Van der Heijden et al., 2020). Through TMA, employees cognitively structure and prioritize time, investing in learning, networking, and skill development to enhance human and social capital (Fugate et al., 2004). TMD translates these cognitive structures into disciplined behavior, maintaining resilience through self-regulatory persistence and recovery after setbacks (Halbesleben et al., 2014). FSC serves as the motivational bridge sustaining future orientation and mitigating impulsivity (Hershfield, 2011; Bartels and Urminsky, 2011). Empirical studies likewise show that future-oriented planning—akin to TMA—predicts adaptability and proactive career behavior under uncertainty (Strauss et al., 2012). Consistent with COR theory, these mechanisms operate synergistically: cognitive structuring (TMA) prevents premature resource loss, behavioral regulation (TMD) ensures efficient time use, and motivational continuity (FSC) promotes reinvestment of psychological and temporal resources. Collectively, they form a cognitive–behavioral–motivational pathway sustaining employability, resilience, and adaptability, extending TMA from financial to organizational domains and illuminating how integrated resource systems support future-oriented functioning in early careers.

## 2 Theoretical background and hypotheses

### 2.1 Time mental accounting and intertemporal choice

In this study, intertemporal choice—the dependent variable—is conceptualized as the relative preference between smaller-sooner and larger-later rewards, and is operationalized through hypothetical monetary trade-offs that capture employees’ temporal decision tendencies. Consistent with established paradigms, this outcome is measured as the frequency of smaller-sooner (SS) reward selections, where higher SS scores indicate stronger present bias and short-term orientation, and lower SS scores reflect greater patience and preference for larger-later rewards. Time Mental Accounting (TMA) stems from Thaler’s theory of mental accounting, which posits that individuals cognitively segregate resources into discrete “accounts” to guide their decisions (Thaler, 1980, 1999). While initially developed in the financial domain, this concept has been extended to time—a finite and non-renewable resource that individuals also allocate across different life domains (Soman, 2001; Zauberman and Lynch, 2005). TMA refers to the psychological process of assigning subjective value and usage rules to time segments categorized into work, leisure, and self-development. These mental accounts shape how individuals perceive opportunity costs and manage trade-offs between short-term rewards and long-term goals (Glimcher et al., 2007). In early-career contexts, TMA may significantly influence novice employees’ decision-making. Individuals entering the workforce must often balance immediate task demands with investments in long-term career development (Stamatogiannakis et al., 2018). Career studies further underscore that early-career workers frequently experience a resource strain between performance pressure and career sustainability, highlighting the need to examine time allocation explicitly through the lens of career development (De Vos et al., 2020). Those who cognitively allocate time to future-oriented accounts—such as career planning or skill development—tend to demonstrate greater deliberation and self-control in their time-related choices (Jachimowicz et al., 2018). Behavioral studies support this perspective, indicating that individuals who strategically structure their time exhibit enhanced self-regulation and are more capable of delaying gratification (Boureau et al., 2015; Ersner-Hershfield et al., 2009). This perspective resonates with the Conservation of Resources (COR) theory, which views time and self-regulatory capacity as valuable resources that individuals strive to protect and invest to achieve future goals (Giurge et al., 2020).

Contemporary research conceptualizes Time Mental Accounting (TMA) as a multidimensional construct encompassing three interrelated facets: loss aversion, mental budgeting, and flexibility (Aimei et al., 2014; Zhang and Huang, 2017). These dimensions parallel financial and mental accounting components and reflect distinct mechanisms of temporal control and prioritization. These facets can be mapped onto different functions within a time-allocation episode rather than being assumed to belong to a single “stage.” Mental budgeting and flexibility mainly describe rule-based account formation and management (budget-setting and cross-account adjustment), whereas loss aversion captures a reference-dependent evaluative function that becomes salient once implicit budgets establish reference points. Accordingly, we discuss them as interlocking components of a broader TMA orientation at the individual-differences level, which also provides the conceptual basis for our SEM operationalization of TMA. Loss aversion reflects individuals’ reference-dependent motivation to avoid wasting time; once implicit time budgets establish a reference point, perceived time waste is experienced as psychologically aversive and irreversible—similar to financial losses (Tversky and Kahneman, 1991). This orientation may promote prudent decision-making by increasing sensitivity to inefficiency and impulsive time use. Mental budgeting refers to the proactive allocation of time toward specific goals, akin to financial budgeting. Individuals with strong mental budgeting tendencies often create psychological “firewalls” to protect long-term objectives from short-term distractions, thus enhancing time structuring and self-control (Bond and Feather, 1988; Jachimowicz et al., 2018). They allocate time segments to work, leisure, or self-development with clear boundaries and foresight. However, classic work on mental budgeting suggests that because budgets cannot perfectly anticipate changing opportunities, budgeting may sometimes constrain reallocation and lead to under-consumption in some categories (e.g., leisure), highlighting a potential tension between budgeting and flexibility (Heath and Soll, 1996).

Flexibility, in contrast, denotes the willingness to reallocate time adaptively in response to changing circumstances. Rather than directly influencing how individuals evaluate near versus distant rewards, flexibility primarily determines how they adjust their schedules and priorities once decisions are made. In contemporary work settings that demand project-based collaboration or rapid adaptation, a certain level of flexibility is practically beneficial, helping employees cope with unexpected demands and shifting deadlines (Archer et al., 2024). However, excessive or unstructured flexibility may weaken adherence to self-imposed budgets, reduce goal persistence and increase susceptibility to task switching or short-term distractions (Leclerc et al., 1995; DeVoe and Pfeffer, 2011). Empirical evidence suggests that flexibility functions more as a situational adaptation mechanism than a stable predictor of intertemporal valuation (Steinmetz et al., 2016). Consequently, flexibility is not expected to exhibit a consistent association with the preference for smaller-sooner rewards in the absence of contextual moderators. Although the multidimensionality of TMA is well established, theoretical and empirical research indicates that these dimensions differ in their influence on intertemporal decision-making (Skwara, 2023). Specifically, loss aversion and mental budgeting have been more consistently linked to enhanced self-regulation and long-term goal pursuit (Bai, 2023; Tversky and Kahneman, 1992). By fostering a heightened awareness of time value and strategic temporal allocation, these facets help individuals resist present bias and prioritize delayed rewards. In contrast, flexibility represents a qualitatively different mechanism. Its effects are highly context-dependent rather than uniformly directional: in stable environments, excessive flexibility may weaken temporal discipline and blur goal boundaries, whereas under high uncertainty, it may facilitate rapid adjustment without necessarily changing intertemporal valuation (Karlsson et al., 1997; Steinmetz et al., 2016). Hence, flexibility is better viewed as a contextual moderator of how individuals apply temporal strategies, not as a direct determinant of present bias or delayed-reward preference. To address these differences, the present study conceptualizes TMA not as a fixed personality trait but as a domain-specific cognitive structuring orientation—an individual’s habitual way of framing and organizing time-related decisions. This conceptualization enables an empirically tractable approach to measuring generalized temporal tendencies without conflating them with immutable personality constructs.

Based on this conceptual differentiation, the present study examines the distinct predictive roles of each TMA subdimension in shaping novice employees’ intertemporal choices. Specifically, we expect loss aversion and mental budgeting to promote future-oriented choices through enhanced temporal discipline and self-control, whereas flexibility—though valuable for situational adaptation—is not expected to exert a consistent effect on intertemporal preference. Clarifying these distinct pathways may contribute to more targeted early-career interventions and refine the theoretical utility of TMA in time-related decision research. Accordingly, we propose the following hypothesis: H1a: Higher loss aversion in TMA is associated with a lower preference for SS rewards. H1b: Higher mental budgeting in TMA is associated with a lower preference for SS rewards. H1c: Flexibility is not significantly associated with intertemporal choice.

### 2.2 The mediating role of future self-continuity

Nevertheless, while TMA provides a valuable cognitive framework for understanding temporal decision tendencies, cognition alone may not fully explain why some employees persist in long-term planning whereas others falter. This limitation underscores the need to examine additional motivational mechanisms that link present actions with future identity, thereby paving the way for introducing Future Self-Continuity (FSC) as a key mediator. Future Self-Continuity (FSC) refers to the perceived psychological connectedness between an individual’s present and future selves (Hershfield, 2011). It captures how individuals perceive their future self as a continuation of the current self rather than a disconnected or unfamiliar identity. Those with high FSC tend to vividly imagine and empathize with their future self, enhancing motivation to consider long-term consequences in daily decision-making. In contrast, individuals with low FSC regard their future self as a psychologically distinct “other,” leading them to discount future outcomes and prioritize immediate gratification (Bartels and Rips, 2010; Bartels and Urminsky, 2011).

A growing body of empirical research highlights the pivotal role of FSC in promoting future-oriented behavior. Individuals with higher FSC are more likely to delay gratification, save for retirement, adopt healthier lifestyles, and resist impulsive temptations (Hershfield et al., 2011; Rutchick et al., 2018). Importantly, FSC is malleable and can be enhanced through targeted interventions—such as visualizing one’s future self or writing letters—which strengthen empathy and identification with future goals (Hershfield et al., 2012; Urminsky, 2017). Within early-career contexts, FSC plays a particularly salient role. Novice employees frequently encounter trade-offs between short-term comforts (e.g., social ease and reduced workload) and long-term investments (e.g., skill development and strategic networking). Those with high FSC are more likely to interpret present efforts as directly contributing to their future professional identity, thereby fostering persistence and goal alignment in the face of delayed rewards (Abrahams et al., 2023).

Theoretically, FSC may be a key psychological mechanism linking Time Mental Accounting (TMA) to intertemporal decision-making. When individuals habitually segment time into future-oriented accounts, they engage in anticipatory thinking that strengthens the continuity between their present and future selves (Yang et al., 2024). For instance, an employee allocating time for additional training reinforces the psychological relevance of future goals, thereby enhancing FSC. Supporting this notion, recent findings suggest that greater temporal awareness is associated with stronger future self-identification and lower temporal discounting (Shen et al., 2023). Drawing on this integrative perspective, we propose that TMA positively predicts FSC, facilitating more prudent intertemporal choices. Novice employees who feel a strong sense of continuity with their future selves are more inclined to prioritize long-term rewards, tolerate short-term discomfort, and make decisions that support sustained career development trajectories. Accordingly, we propose the following hypothesis: H2: FSC mediates the relationship between TMA and intertemporal choice.

### 2.3 The mediating role of time management disposition

Time Management Disposition (TMD) refers to an individual’s enduring tendency to perceive time as a valuable resource, monitor its use, and allocate it efficiently toward meaningful goals (Claessens et al., 2007; Fu et al., 2025). It reflects a trait-like inclination encompassing time awareness, planning, prioritization, and resistance to procrastination or distraction. Individuals with high TMD typically establish clear goals, structure daily routines, and maintain adherence to timelines, while those with low TMD often struggle with time wastage, disorganization, and impulsive task switching (Britton and Tesser, 1991; Macan, 1994).

Conceptually and empirically, TMD is closely linked to self-regulation and executive functioning, as effective time management requires impulse control, resistance to procrastination, and the consistent implementation of planned activities over time (Aeon and Aguinis, 2017; Duckworth et al., 2014). Although conceptually related to the mental budgeting facet of Time Mental Accounting, TMD emphasizes behavioral enactment—how individuals translate cognitive evaluations of time into sustained patterns of goal-directed action, encompassing both the completion of immediate tasks and the pursuit of broader objectives. In organizational settings, individuals with strong TMD are more likely to meet deadlines, balance work and life demands, and dedicate time to strategically important yet non-urgent tasks such as skill development or mentorship (Chiaburu and Marinova, 2005; König and Kleinmann, 2007). Moreover, TMD has been positively associated with a range of performance outcomes in both academic and occupational domains, including higher productivity, improved stress management, and enhanced subjective wellbeing (Aeon et al., 2021; Saloviita, 2013). Beyond its trait-like description, TMD has been conceptualized in prior research as a process-oriented self-regulatory mechanism that translates cognitive evaluations of time into concrete behavioral strategies (Aeon and Aguinis, 2017; Claessens et al., 2007) This perspective highlights TMD not merely as a background condition, but as an active pathway through which individuals convert abstract time perceptions into consistent patterns of goal-directed action. In this sense, TMD serves as a mediator by explaining how cognitive structuring of time (TMA) is enacted in daily behavior, ultimately shaping intertemporal decision outcomes.

For the present study, TMD is a behavioral mechanism that supports intertemporal decision-making. It enables individuals to resist the appeal of immediate, smaller rewards by focusing on long-term objectives and allocating time strategically toward delayed outcomes (Aeon and Aguinis, 2017; Zimbardo and Boyd, 2014). For example, a high-TMD novice employee may invest additional time in professional training or skill-building activities despite short-term inconvenience, having internalized the value of long-term growth. Furthermore, TMD facilitates what has been termed “temporal self-alignment”—the alignment of present actions with anticipated future benefits—a core aspect of prudent intertemporal decision-making (Sirois, 2014). Those with strong TMD are also more adept at converting intentions into consistent action over time, bridging the well-documented intention—behavior gap in goal pursuit (Steel and König, 2006). Taken together, TMD is not merely a personality trait but a dynamic self-regulatory capacity that enhances temporal discipline and goal consistency. This study proposes that TMD mediates the relationship between time-mental accounting and intertemporal choice by enabling individuals to translate long-term intentions into structured, time-conscious behavior. Accordingly, we propose the following hypothesis: H3: TMD mediates the relationship between TMA and intertemporal choice.

### 2.4 Integrative framework and hypotheses

**Figure 1:**
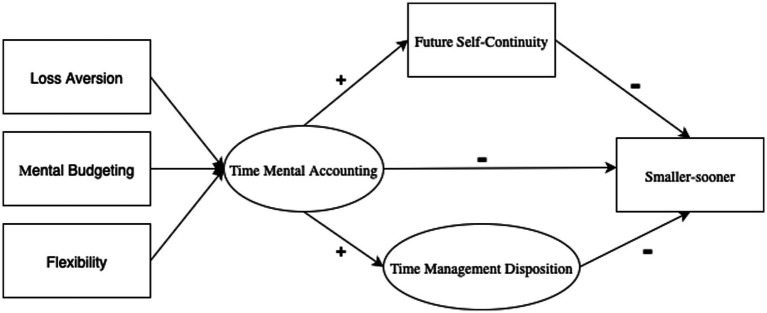
The proposed parallel mediation model.

Building on the preceding theoretical foundation, this study advances an integrative framework grounded in Conservation of Resources (COR) theory (Hobfoll, 1989; Halbesleben et al., 2014) to explain how novice employees make intertemporal choices through the coordinated use of cognitive, behavioral, and motivational resources. COR theory posits that individuals strive to acquire, protect, and invest valuable resources to prevent loss and promote growth. Within this framework, time is conceptualized not merely as a chronological unit but as a finite psychological resource whose allocation reflects one’s capacity to organize, regulate, and sustain goal-directed effort under competing short- and long-term demands. Accordingly, we propose that intertemporal decision-making reflects a process of resource orchestration involving three interrelated mechanisms: Time Mental Accounting (TMA) as a cognitive resource, Time Management Disposition (TMD) as a behavioral resource, and Future Self-Continuity (FSC) as a motivational resource. Prior research has typically examined these mechanisms in isolation—cognitive structuring of time (Wu and He, 2012), behavioral regulation of time use (Aeon and Aguinis, 2017), or motivational continuity with the future self (Hershfield, 2011; Yang et al., 2024)—thus overlooking how they interact to sustain adaptive self-regulation. Recent reviews have explicitly called for integrative models that combine cognitive, behavioral, and identity-based perspectives to capture the multidimensional nature of temporal decision-making (Duckworth et al., 2014; Aeon and Aguinis, 2017; see Figure 1).

Within this framework, TMA provides the cognitive scaffolding through which individuals assign subjective value to time, evaluate opportunity costs, and mentally budget their temporal resources. It is operationalized as a multidimensional construct encompassing loss aversion, mental budgeting, and flexibility (Antonides and de Groot, 2022; Singh and Nandan, 2024). These dimensions mirror financial mental accounting mechanisms and display differential links to self-regulatory outcomes: loss aversion and mental budgeting are typically associated with stronger long-term orientation, whereas flexibility may promote adaptive adjustment under situational uncertainty. For theoretical clarity, TMA is conceptualized as a multidimensional construct comprising three facets—loss aversion, mental budgeting, and flexibility—but these dimensions are analyzed separately to capture their distinct predictive contributions to intertemporal choice. The influence of TMA on intertemporal choice operates indirectly through two complementary pathways. TMD represents behavioral self-regulation—the structured implementation of time use that converts cognitive plans into effective action—whereas FSC captures motivational self-integration, reflecting a sustained psychological connection to one’s future self that preserves goal alignment over time. Together, TMD and FSC serve as mediating mechanisms that transform cognitive framing into behavioral and motivational outcomes, consistent with process models of self-control (Thaler and Shefrin, 1981; Duckworth et al., 2014) and COR theory’s principle that multiple resources must operate in synergy to prevent depletion and foster resilience.

By situating intertemporal choice research at the intersection of behavioral economics, temporal psychology, and self-regulation theory, this integrative framework provides a cross-disciplinary and theoretically unified model. It emphasizes that intertemporal choice is not simply an economic trade-off but a resource-based decision system in which cognitive structuring (TMA) enables efficient behavioral execution (TMD) and sustained motivational investment (FSC). The model is particularly relevant for early-career employees, whose limited experience and resource reserves heighten vulnerability to short-term bias. Under the COR perspective, cultivating cognitive, behavioral, and motivational resources in coordination becomes essential for maintaining adaptive functioning and career sustainability (Bizhong and Xiaojun, 2022).

In summary, this framework positions TMA as the cognitive foundation of temporal decision-making, whose influence unfolds through the behavioral pathway of TMD and the motivational pathway of FSC. Given the cross-sectional design, the mediation relationships tested in this study should be interpreted as associations rather than definitive causal pathways. To enhance robustness, we tested an alternative reverse model (TMD/FSC → TMA → intertemporal choice), and the results showed inferior model fit compared to the hypothesized model, supporting the theoretical directionality proposed. By embedding these mechanisms within the Conservation of Resources (COR) theory, the model provides an integrated explanation of how novice employees allocate and reinvest their temporal and psychological resources to balance short- and long-term demands. This theoretical structure directly underpins the three hypotheses (H1–H3) introduced earlier, each corresponding to one pathway within the dual-mediation model. H1a–H1c specify how the three facets of Time Mental Accounting (loss aversion, mental budgeting, and flexibility) are expected to be associated with preferences for smaller-sooner versus larger-later rewards; H2 states that Future Self-Continuity mediates the association between TMA and intertemporal choice; and H3 states that Time Management Disposition mediates the association between TMA and intertemporal choice.

## 3 Materials and methods

### 3.1 Participants and procedure

The study targeted novice employees, defined as individuals with no more than 3 years of full-time work experience. Participants were recruited via Credamo, a professional online survey platform, and a total of 700 respondents from all 32 provincial-level regions of China completed the questionnaire using mobile phones, computers, or other digital devices. To ensure data quality, stringent exclusion criteria were applied: respondents were excluded if they (a) exceeded the work experience threshold (i.e., were unemployed or had more than 3 years of full-time employment), (b) failed an attention-check item (“I feel time passes too slowly”—correct answer: “completely disagree”), (c) completed any major scale in less than 1 s per item (indicating inattentive responding), or (d) exhibited straight-lining response patterns (e.g., selecting the same option across consecutive items). After excluding 103 invalid responses, the final analytic sample comprised 597 valid cases, yielding an effective response rate of 85.16%. The average age of the participants was 25.3 years (SD = 2.8), with a gender distribution of 60.14% male (*N* = 359) and 39.86% female (*N* = 238). Regarding marital status, 41.71% were single, 28.64% in a relationship, and 29.65% married. Regarding educational attainment, 77.89% held a bachelor’s degree, 8.54% had a master’s or doctoral degree, 9.72% had an associate degree, and 3.85% had completed high school or below. Monthly income ranged from below 2,000 RMB to above 10,000 RMB, with the largest segment earning 4,000–6,000 RMB (43.71%), followed by 6,000–10,000 RMB (29.64%) and 2,000–4,000 RMB (17.09%). Participants were employed across a range of sectors, including the private sector (41.20%), public service or civil institutions (24.96%), state-owned enterprises (20.94%), foreign-owned firms (6.37%), and self-employment (6.53%). Most respondents had 1–2 years of work experience (39.36%), followed by 0–1 year (21.60%) and 2–3 years (39.04%). This distribution reflects the early stages of career development and aligns with the study’s target population. The sample’s wide variation in region, industry, income level, and educational background enhances the external validity and generalizability of the findings to the broader population of young professionals in China. Participant demographic characteristics are presented in Table 1.

**Table 1:** Socio-demographic information of participants (*n* = 597).

Characteristic	N	%
Gender		
Male	359	60.14
Female	238	39.86
Age	*M* = 26.34	SD = 2.89
Marital status		
Single	249	41.71
In a relationship	171	28.64
Married	177	29.65
Educational attainment		
High school/Vocational school or below	23	3.85
Associate degree	58	9.72
Bachelor’s degree	465	77.89
Master’s or doctoral degree	51	8.54
Monthly income		
Below 2,000 RMB	19	3.19
2000–4,000 RMB	102	17.09
4,000–6,000 RMB	261	43.71
6,000–10,000 RMB	177	29.64
Above 10,000 RMB	38	6.37
Occupation type		
State-owned enterprise	125	20.94
Public sector/Civil service	149	24.96
Private sector	246	41.20
Foreign-owned enterprise	38	6.37
Self-employed	39	6.53
Years of employment		
0–1 year	129	21.60
1–2 years	235	39.36
2–3 years	233	39.04

All procedures were approved by the Ethics Committee of Southwest University (Approval No. H24117), and informed consent was obtained from all participants prior to survey administration. The study was conducted by the ethical principles of the Declaration of Helsinki, and all data were collected anonymously in compliance with institutional data protection standards.

### 3.2 Measures

#### 3.2.1 Demographic information

Demographic information was collected using several asking participants about their gender, marital status, educational attainment, average monthly income, occupation type and Years of Employment.

#### 3.2.2 Time Mental Accounting Scale

The Time Mental Accounting Scale was used to measure perceived time mental accounting (Yang, 2009). It is a 9-item scale with three dimensions: loss aversion (3 items, e.g., “If I waste time on an activity, I will endeavor to save time on other activities”), mental budgeting (3 items, e.g., “I generally ensure a proper balance between work time and leisure time”), and flexibility (3 items, e.g., “The time I allocate to different activities is flexibly adjusted based on the needs”). Here, mental budgeting reflects setting and maintaining planned time budgets across major domains (e.g., a work–leisure budget), whereas flexibility reflects reallocating time across activities when situational demands change. Responses were recorded using a six-point Likert scale, ranging from 1 (“strongly disagree”’) to 6 (“strongly agree”). The reliability of the scale was confirmed with a Cronbach’s alpha of 0.7624 in this study.

#### 3.2.3 Future Self-Continuity Scale

The Future Self-Continuity Scale was used to assess the similarity between current and future selves (Ersner-Hershfield et al., 2009). As such, the index of future self-continuity is measured by 2 questions on a 7-point scale marked at each point by two circles that ranged from depicting no overlap to depicting almost complete overlap. The first question asks participants to select the circle pair that best described how similar they felt to a future self 10 years from now on a scale ranging from 1 (“not similar at all”) to 7 (“completely similar”). The higher scores reflected the more continuity with one’s future self. The test–retest reliability over a two-week interval was found to be high (Hershfield et al., 2012), with coefficients of 0.79 for similarity and 0.80 for connectedness. It has been indicated that the scale possesses distinct discriminant validity from measures of related constructs, such as uncertainty about the future, future preferences, and perceived general life changes (Bartels and Urminsky, 2011). Although the measure comprises only two visual items, its psychometric robustness and predictive validity have been reliably and repeatedly demonstrated across behavioral and decision-making studies, establishing it as a widely recognized operationalization of future self-continuity (Rutchick et al., 2018).

#### 3.2.4 Time Management Disposition Scale

The Time Management Disposition Scale was used to measure perceived time management disposition (Yong-Hong and Xi-Ting, 2011). It is a 45-item scale with three dimensions: time value (29 items, e.g., “Time is the most precious resource”), time monitoring concept (8 items, e.g., “I typically set long-term goals”), and time efficacy (8 items, e.g., “I am confident in my ability to use time well”). Items are rated on a 6-point scale from “strongly disagree” to “strongly agree,” with higher scores indicating higher levels of time management disposition. In the current study, confirmatory factor analysis (CFA) substantiated the construct validity of the scale, yielding favorable metrics: χ^2^/df = 2.35, CFI = 0.92, SRMR = 0.037, RMSEA = 0.046. These values affirm the structural integrity and suitability of the scale for this research.

#### 3.2.5 Intertemporal choice preference

The dependent variable, intertemporal choice preference, was operationalized using a set of binary choice tasks that contrasted smaller-sooner (SS) rewards with larger-later (LL) rewards—a widely validated paradigm in intertemporal decision-making research (Zhou et al., 2024). Although the present task involved monetary trade-offs, this paradigm has been widely validated as a proxy for temporal decision-making because both time and money rely on similar discounting processes and cognitive mechanisms (Peters and Büchel, 2011; Zauberman and Lynch, 2005). To ensure sensitivity across participants and avoid floor or ceiling effects, a pilot study involving 332 novice employees was conducted to identify approximate indifference points—reward combinations where participants were roughly equally likely to choose between SS and LL options. Based on the pilot results, eight decision scenarios were selected for the primary survey. Each scenario presented an immediate monetary reward (ranging from ¥50 to ¥7,000) versus a larger delayed reward available after a delay ranging from 1 month to 4 years. For example, one scenario asked participants to choose between “Receive ¥67 now or ¥100 in 30 days,” and another presented the choice “Receive ¥7,173 in 2 years or ¥10,000 in 4 years.” Participants were required to make a selection in each of the eight scenarios. For each participant, an SS score was calculated by counting the number of instances in which the smaller-sooner reward was chosen over the larger-later one. This score ranged from 0 (indicating a consistent preference for delayed rewards and thus greater patience) to 8 (indicating a consistent preference for immediate rewards and thus stronger present bias). A higher SS score reflects a stronger short-term preference, whereas lower scores indicate a greater willingness to delay gratification. This continuous SS score was the primary outcome variable, capturing individual differences in intertemporal decision-making tendencies.

### 3.3 Data analysis

All statistical analyses were conducted using IBM SPSS Statistics 26.0 and AMOS 24.0. Descriptive statistics and Pearson correlation coefficients were computed to examine associations among the key variables: Time Mental Accounting (TMA), Time Management Disposition (TMD), Future Self-Continuity (FSC), and short-term preference (SS score). Given the multidimensional nature of TMA, its three subdimensions—loss aversion, mental budgeting, and time flexibility—were also examined separately to preliminarily explore their differential associations with the mediators and the outcome variable. To test the hypothesized mediation effects (H2 and H3), structural equation modeling (SEM) was conducted in AMOS, with TMA specified as a latent construct indicated by its three subdimensions. The model included two parallel mediators (TMD and FSC), which were allowed to covary, and one outcome variable (SS score). Age, gender, and education level were included as control variables. Model fit was evaluated using conventional indices: the chi-square to degrees of freedom ratio (χ^2^/df), Comparative Fit Index (CFI), Root Mean Square Error of Approximation (RMSEA), and Standardized Root Mean Square Residual (SRMR). Indirect effects were tested using bias-corrected bootstrapping (5,000 resamples), with statistical significance determined by 95% confidence intervals that excluded zero. All tests were two-tailed with an alpha level of 0.05.

## 4 Results

### 4.1 Analysis of common methodological bias

To examine potential common method bias (CMB), both exploratory and confirmatory statistical procedures were conducted following established recommendations (Podsakoff et al., 2003; Fuller et al., 2016). First, Harman’s single-factor test was performed using unrotated exploratory factor analysis. The results showed that the first unrotated factor accounted for 21.82% of the total variance, which is well below the conservative threshold of 40%, indicating that no single factor dominated the variance. Moreover, 14 factors had eigenvalues greater than one, suggesting that the data structure was not excessively influenced by a single common source.

To further assess CMB beyond Harman’s test, we conducted a conceptual check using theoretically unrelated demographic variables. Specifically, correlations between education level, occupation type, and the focal constructs were examined. All correlations were weak and nonsignificant (|*r*| < 0.10), supporting the conclusion that common method variance was unlikely to substantially bias the observed relationships. Taken together, these findings suggest that common method bias was not a serious concern in this study.

### 4.2 Descriptive statistics and correlations

A correlation analysis was conducted to examine the relationships among the primary variables of interest: time mental accounting (TMA), time management disposition (TMD), future self-continuity (FSC), and short-term preference (SS). TMA was conceptualized as comprising three subdimensions—loss aversion, flexibility, and mental budgeting—while TMD included time value, time monitoring, and time efficacy. Results showed that both loss aversion and mental budgeting were significantly positively correlated with TMD (*r* = 0.51 and *r* = 0.65, respectively, *p* < 0.001) and with FSC (*r* = 0.15 and *r* = 0.14, respectively, *p* < 0.001), suggesting that valuing time and planning time are associated with better time management and a stronger connection to one’s future self. In contrast, time flexibility showed no significant associations with TMD or FSC. Regarding the outcome variable, short-term preference was negatively correlated with loss aversion (*r* = −0.20, *p* < 0.001), mental budgeting (*r* = −0.17, *p* < 0.001), TMD (*r* = −0.22, *p* < 0.001), and FSC (*r* = −0.19, *p* < 0.001), indicating that individuals who are more strategic in how they value and allocate their time are less likely to make impulsive decisions. No significant correlation was found between flexibility and short-term preference. Given these null associations, time flexibility was not included in the subsequent mediation analyses. These findings provide preliminary support for the hypothesized relationships and highlight the differential roles of TMA subdimensions in intertemporal choice (see Table 2). Prior to conducting the mediation analyses, diagnostic tests were performed to examine potential multicollinearity and conceptual overlap between the two mediators—Time Management Disposition (TMD) and Future Self-Continuity (FSC). The correlation was modest (*r* = 0.22, *r*^2^ = 0.048), indicating limited shared variance. Variance inflation factor (VIF = 1.013) and tolerance values (0.987) further confirmed the absence of multicollinearity (VIF < 5, tolerance > 0.10). These results suggest that TMD and FSC are statistically distinct constructs, thereby supporting discriminant validity between the two mediators.

**Table 2:** Correlations, means, and standard deviations of the study variables.

Variable	M±	SD	1	2	3	4	5	6	7	8
Loss aversion	14.42	2.14	–							
Flexibility	13.10	2.12	0.177	–						
Mental budgeting	14.41	2.24	0.452**	0.113**	–					
Time management disposition	175.63	16.84	0.512**	0.024	0.648**	–				
Time value	34.35	3.36	0.487**	0.190**	0.444**	0.689**	–			
Time monitoring concept	107.86	11.56	0.438**	−0.031	0.620**	0.968**	0.526**	–		
Time efficacy	33.42	3.78	0.508**	0.031	0.596**	0.880**	0.574**	0.788**	–	
Future self-continuity	4.72	1.35	0.154**	−0.054	0.144**	0.219**	0.138**	0.210**	0.209**	–
Smaller-sooner choices	4.21	2.17	−0.200**	−0.007	−0.166**	−0.217**	−0.194**	−0.188**	−0.220**	−0.187**

*p < 0.05, **p < 0.01, ***p < 0.001, M, mean, SD, standard deviation.

### 4.3 The mediating effects of TMD and FSC on the relationship between time mental accounting and intertemporal choices

**Figure 2:**
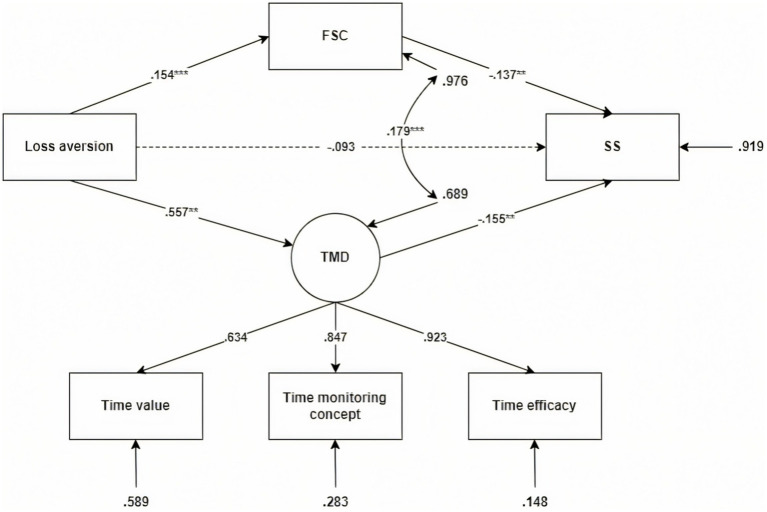
Mediation model: The roles of time management disposition and future self-continuity in the relationship between loss aversion and intertemporal choice. FSC, future self-continuity; TMD, time management disposition; SS, smaller-sooner choices. χ^2^ = 34.055, df = 6, χ^2^/df = 5.68, CFI = 0.975, TLI = 0.954, SRMR = 0.029, RMSEA = 0.078. The model explained 41% of the variance in TMD (*R*^2^ = 0.41), 29% in FSC (*R*^2^ = 0.29), and 37% in SS (*R*^2^ = 0.37), indicating satisfactory explanatory power.

**Figure 3:**
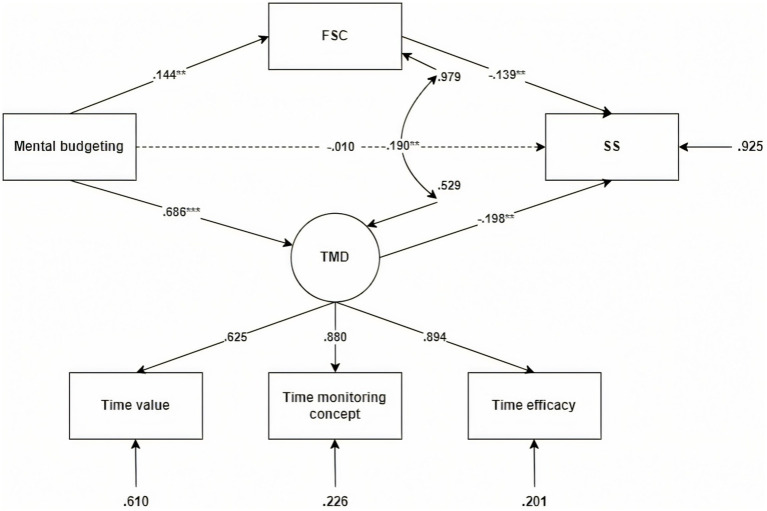
Mediation model: The roles of time management disposition and future self-continuity in the relationship between mental budgeting and intertemporal choice. χ^2^ = 11.045, df = 6, χ^2^/df = 1.84, CFI = 0.996, TLI = 0.987, SRMR = 0.013, RMSEA = 0.038. The model explained 43% of the variance in TMD (*R*^2^ = 0.43), 33% in FSC (*R*^2^ = 0.33), and 39% in SS (*R*^2^ = 0.39), indicating excellent explanatory power and model fit.

To elucidate the psychological mechanisms linking Time Mental Accounting (TMA) to intertemporal decision-making, we tested two parallel mediation models focusing on key TMA subdimensions: loss aversion and mental budgeting. Time Management Disposition (TMD) and Future Self-Continuity (FSC) were specified as parallel mediators in both models. Structural equation modeling (SEM) was employed for path analysis, and model fit indices indicated good fit in both cases (e.g., CFI > 0.97, RMSEA < 0.08; see Figures 2, 3), supporting the adequacy of the proposed structures.

In the loss aversion model, the direct effect of loss aversion on short-term preference was non-significant (*β* = −0.093, 95% CI [−0.179, 0.007]), while both indirect effects were significant. Specifically, the mediation path via TMD yielded *β* = −0.021 (95% CI [−0.042, −0.007], *p* < 0.05), and via FSC *β* = −0.086 (95% CI [−0.146, −0.034], *p* < 0.01), indicating complete mediation through both behavioral regulation and psychological continuity mechanisms (see Table 3). In the mental budgeting model, the direct effect of mental budgeting on short-term preference was also non-significant (*β* = −0.010, 95% CI [−0.109, 0.086], *p* = 0.845), whereas indirect effects via TMD (*β* = −0.020, 95% CI [−0.039, −0.007], *p* < 0.05) and FSC (*β* = −0.136, 95% CI [−0.222, −0.063], *p* < 0.001) were significant, again indicating complete mediation (see Table 4).

**Table 3:** Direct and indirect effects in the mediation model with loss aversion as predictor.

Variable pathway	β	Bootstrap SE	*p*	95% CI lower	95% CI upper
Indirect effects					
Loss aversion→SS	−0.093	0.047	0.049	−0.179	0.007
Indirect effects					
Loss aversion→TMD → SS	−0.021	0.009	0.013	−0.042	−0.007
Loss Aversion→FSC → SS	−0.086	0.029	0.003	−0.146	−0.034

**Table 4:** Direct and indirect effects in the mediation model with loss aversion as predictor.

Variable pathway	β	Bootstrap SE	*p*	95% CI lower	95% CI upper
Indirect effects					
Mental budgeting→SS	−0.010	0.051	0.845	−0.109	0.086
Indirect effects					
Mental budgeting→TMD → SS	−0.020	0.008	0.016	−0.039	−0.007
Mental budgeting→FSC → SS	−0.136	0.040	0.001	−0.222	−0.063

Notably, the strength of the mediation paths varied by predictor: the FSC pathway was more pronounced in the loss aversion model, suggesting that perceiving time as non-recoverable enhances identification with the future self; in contrast, the TMD pathway was more salient in the mental budgeting model, underscoring the role of structured time allocation in facilitating behavioral self-regulation. These findings support Hypotheses 2 and 3 robustly, demonstrating that TMA fosters future-oriented decision-making among novice employees not through direct influence but by enhancing every day time management practices and strengthening future-oriented self-construal. These results suggest that interventions promoting time structuring and long-term self-awareness may help mitigate impulsive choices during early career development.

## 5 Discussion

### 5.1 Theoretical and practical implications

This study investigated how Time Mental Accounting (TMA) relates to intertemporal decision-making among novice employees and examined the mediating roles of Time Management Disposition (TMD) and Future Self-Continuity (FSC). The findings provide strong empirical support for the proposed dual-mediation model, offering novel insights into how cognitive frameworks, behavioral competencies, and temporal identity jointly shape time-related decision preferences in early-career individuals. Consistent with theoretical perspectives on temporal cognition and self-regulation (Duckworth et al., 2018; Ersner-Hershfield et al., 2009; Hall and Fong, 2007), our results show that TMA—particularly the subdimensions of mental budgeting and loss aversion—is associated with intertemporal decision-making through two distinct psychological pathways: behavioral regulation (TMD) and future-oriented identity (FSC). Notably, neither subdimension significantly influenced preference for smaller-sooner (SS) rewards when mediators were included, indicating a complete mediation structure. Although the standardized indirect effects ranged from −0.02 to −0.13, such magnitudes are consistent with those typically observed in mediation models of self-regulation and identity processes (Duckworth et al., 2018; Rutchick et al., 2018). Given that intertemporal preferences are shaped by multiple contextual and dispositional factors, even small effects may accumulate through repeated daily choices, influencing long-term career outcomes (Aeon et al., 2021). These findings therefore highlight that modest gains in time management or future-self connectedness can produce meaningful improvements in self-regulatory alignment over time.

These findings align with dual-process models of self-control, such as the planner–doer framework (Thaler and Shefrin, 1981), which emphasizes the transformation of abstract planning into concrete self-regulatory action and motivational commitment to guide long-term choices. Further analysis revealed functional differentiation between the two mediation pathways: loss aversion was primarily transmitted through FSC, whereas TMD predominantly mediated the effect of mental budgeting. This pattern suggests that distinct temporal cognitions engage complementary self-regulatory systems. Within the COR framework, such differentiation may represent alternative pathways of resource investment: structured time budgeting functions as a behavioral strategy for conserving regulatory resources through planning and control (Hall and Fong, 2007), whereas stronger future self-continuity acts as a motivational investment that reinforces the perceived value of future outcomes and prevents premature depletion of psychological energy (Hershfield, 2011). Together, these mechanisms illustrate a dynamic resource process in which cognitive framing of time facilitates the conservation and reinvestment of self-regulatory and motivational resources—thereby sustaining adaptive functioning and long-term alignment. This differentiation also mirrors dual-process models of decision-making (Kahneman, 2011), where loss aversion reflects an intuitive, affect-driven orientation toward avoiding temporal losses (System 1), while mental budgeting embodies deliberative, rule-based planning processes characteristic of System 2 thinking. Together, these findings clarify how affective and analytic temporal mechanisms jointly guide intertemporal control. These interpretations echo prior findings that psychological connectedness to the future self-promotes delayed gratification (Rutchick et al., 2018) and complement time management research highlighting the role of planning and prioritization in effective self-regulation (Aeon et al., 2021; Claessens et al., 2007). Significantly, the results extend mental accounting theory by demonstrating that principles traditionally applied to financial decision-making—such as mental budgeting and loss aversion (Thaler, 1999)—are equally relevant in time.

By contrast, the third TMA dimension, time flexibility, showed no meaningful association with either mediators or outcomes. This null pattern is theoretically compatible with the budgeting–flexibility trade-off emphasized in the mental accounting literature: while stronger budgeting boundaries can protect planned allocations, they can also constrain reallocation across accounts (including leisure), yielding countervailing forces that may offset each other at the aggregate level (Heath and Soll, 1996; Steinmetz et al., 2016). Future work might examine whether moderate flexibility promotes adaptability without undermining goal pursuit or whether temporal rigidity is generally more conducive to effective long-term planning. The mediating role of TMD reinforces its status as a central self-regulatory resource. While previous studies have primarily associated TMD with productivity and wellbeing, our findings extend its relevance to strategic temporal decision-making. TMD functions not merely as a daily efficiency skill but as a behavioral mechanism through which individuals allocate and preserve regulatory resources for long-term goals. Likewise, FSC emerges as a dynamic and cultivable motivational resource that strengthens the continuity between present and future selves, mitigating impulsive tendencies and enhancing persistence (Bartels and Rips, 2010; Hershfield et al., 2011). These findings support an integrative framework that bridges behavioral economics, temporal psychology, and self-regulation theory. Integrating these findings within the Conservation of Resources (COR) framework offers a deeper theoretical interpretation. COR theory posits that individuals strive to acquire, protect, and invest valued resources to prevent depletion and foster adaptive functioning (Hobfoll, 1989). In this context, TMA, TMD, and FSC can be conceptualized as complementary components of a temporal resource system: cognitive mechanisms (TMA) provide the structure for resource allocation; behavioral strategies (TMD) regulate how resources are enacted and conserved; and motivational factors (FSC) ensure that future-oriented goals retain psychological value. Guided by this COR-based resource-system view, we treat the dual-mediation specification as conceptually central because it identifies the behavioral and motivational resource pathways (TMD and FSC) through which time mental accounting is associated with intertemporal choice, whereas moderation would primarily speak to boundary conditions. Together, these processes reflect a dynamic cycle of conservation and reinvestment, whereby individuals seek to minimize loss spirals and maintain long-term equilibrium in their cognitive and emotional energy. This resource-based interpretation not only grounds the dual mediation model within a broader theoretical ecology but also aligns intertemporal decision-making with the fundamental principles of resource preservation and adaptive resilience.

Whereas traditional models of intertemporal choice have emphasized discount functions or impulsivity parameters (Frederick et al., 2002; O’Donoghue and Rabin, 1999), our model shows that an interplay between time-based cognition, self-regulatory capacity, and future-oriented identity shapes long-term preferences. Notably, TMA alone was insufficient to reduce short-term bias unless accompanied by high levels of TMD and FSC. This supports contemporary motivational-control perspectives (Duckworth et al., 2018), which argue that multiple psychological systems must co-activate to produce behavioral change. Theoretically, this study underscores the need to move beyond simplistic patience–impulsivity dichotomies and toward more nuanced frameworks that integrate cognitive appraisal (TMA), regulatory functioning (TMD), and identity-based motivation (FSC). Such models offer promising directions for future research exploring how individuals form sustainable goal-pursuit strategies in complex environments. Potential extensions may examine moderators such as conscientiousness (Waldeyer et al., 2022), cultural values (Hofstede, 2001), or emotion regulation strategies that shape the TMA–TMD/FSC–decision pathway (Yoon and Jung, 2025). Conscientiousness may strengthen the translation of mental budgeting into effective time management, consistent with findings that highly conscientious individuals benefit more from time-management and effort regulation strategies (Waldeyer et al., 2022). Cultural values such as long-term orientation or collectivism are likely to reinforce future self-continuity, whereas short-term or individualistic orientations may weaken it (Hofstede, 2001). Moreover, emotion regulation strategies like cognitive reappraisal may help individuals resist immediate temptations and align behavior with future-oriented goals (Yoon and Jung, 2025). This study offers robust empirical evidence for a dual-mediation model in which TMA influences intertemporal preferences among novice employees via self-regulation and temporal identity mechanisms. These results highlight the need for interventions that enhance the cognitive structuring of time and cultivate future-oriented thinking to support long-term success in the workplace.

### 5.2 Limitations and future directions

Despite the valuable insights this study contributes, several limitations warrant consideration. First, the cross-sectional nature of the data constrains causal inference. Although the proposed mediation pathways—from Time Mental Accounting (TMA) through Time Management Disposition (TMD) and Future Self-Continuity (FSC) to intertemporal choice—are theoretically grounded, the possibility of reverse or reciprocal causality cannot be entirely excluded. Longitudinal studies or experimental interventions—such as time-management training or future visualization techniques—could offer stronger causal evidence and actionable implications (Cook and Campbell, 1979).

Second, the sample was restricted to novice white-collar employees in China, a context characterized by a culturally embedded long-term orientation (Zhang and He, 2024). This cultural predisposition may have amplified the salience of future-oriented constructs such as FSC, potentially inflating the strength of observed associations. Future research should examine the model across cultures and occupational strata to enhance external validity, including blue-collar employees, mid-career professionals, and individuals from societies with weaker future orientation or greater individualism.

Third, the operationalization of intertemporal choice relied on hypothetical monetary trade-offs, which may not fully capture real-world behaviors. Although such measures are standard in experimental paradigms, future studies should incorporate ecologically valid behavioral metrics (e.g., actual training investments, project commitment levels, or delay-of-reward behaviors) across diverse health, leisure, or career development domains. This would allow a more comprehensive assessment of intertemporal reasoning in everyday decision-making.

Fourth, several measurement considerations warrant mention. FSC was assessed using the two-item overlapping-circle task. Although this measure is concise, theoretically grounded, and effectively captures perceived self–future connectedness, its brevity may compromise statistical stability in mediation analyses. Future research should consider employing multi-item variants or extended versions to enhance psychometric robustness (Hershfield et al., 2011). TMD was measured with a 45-item instrument capturing the multidimensional structure of temporal self-regulation. While such comprehensiveness may marginally inflate path coefficients due to shared-method variance, it ensures theoretical coverage and construct integrity. Future studies could examine whether shorter validated scales yield comparable structural patterns with greater efficiency (Aeon and Aguinis, 2017). Intertemporal choice was operationalized through hypothetical monetary trade-offs—standard in behavioral economics yet limited in ecological validity—suggesting that future research should incorporate behavioral indicators such as actual time investments, task persistence, or delay-of-reward behaviors to better approximate real-world decision-making.

Finally, although TMD and FSC were modeled as parallel mediators, their potential interactive effects were not explored. It remains an open question whether strong future self-continuity can compensate for weak time-management habits or if their joint presence is necessary for promoting far-sighted choices. In addition, TMD and FSC may be shaped by influences beyond time mental accounting (e.g., training experiences and stable individual differences), and educational attainment may serve as a meaningful boundary condition; future research could model these factors directly and test moderated mediation to examine whether the relative strength of the TMD versus FSC pathways varies across education levels. Moreover, the contextual role of time flexibility warrants further attention. Given that flexibility was not significantly associated with intertemporal choice in the present model, future studies could examine whether moderate flexibility enhances adaptability without undermining long-term goal pursuit, or whether it interacts with TMD and FSC under specific work conditions such as high job autonomy or task uncertainty. Testing these interaction and contextual effects would provide a more nuanced understanding of how behavioral regulation, temporal identity, and situational adaptability jointly shape intertemporal decision-making.

## 6 Conclusion

This study employed a parallel mediation model to investigate how Time Mental Accounting (TMA) influences intertemporal decision-making among novice employees, with Time Management Disposition (TMD) and Future Self-Continuity (FSC) serving as independent mediators. The results demonstrate that individuals with higher levels of TMA tend to exhibit more effective time management and stronger psychological connectedness to their future selves, both independently associated with a greater preference for long-term over immediate rewards. These findings highlight the pivotal role of time-related cognitive frameworks in shaping early-career decision strategies. From a practical standpoint, employees may benefit from time-budgeting and future-self visualization exercises, managers could foster mentoring and goal reviews, and organizations may incorporate time-management training into onboarding programs. Together, such initiatives may enhance young employees’ capacity for strategic planning, delay of gratification, and sustained goal pursuit—key attributes for long-term professional development. While these initiatives align with the mechanisms identified in this study, their effectiveness should be interpreted cautiously given the cross-sectional design. Future experimental or longitudinal research is needed to establish causal links and evaluate how such interventions translate into sustainable career development.

## Data Availability

The datasets presented in this study can be found in online repositories. The names of the repository/repositories and accession number(s) can be found in the article/Supplementary material.
